# mRNA in situ hybridization exhibits unbalanced nuclear/cytoplasmic dystrophin transcript repartition in Duchenne myogenic cells and skeletal muscle biopsies

**DOI:** 10.1038/s41598-023-43134-6

**Published:** 2023-09-24

**Authors:** Maria Sofia Falzarano, Martina Mietto, Fernanda Fortunato, Marianna Farnè, Fernanda Martini, Pierpaolo Ala, Rita Selvatici, Francesco Muntoni, Alessandra Ferlini

**Affiliations:** 1 Department of Medical Sciences, Unit of Medical Genetics, University of Ferrara, Ferrara, Italy; 2 Department of Medical Sciences, Section of Experimental Medicine, University of Ferrara, Ferrara, Italy; 3 Dubowitz Neuromuscular Centre and National Institute for Health Research, Great Ormond Street Institute of Child Health, Biomedical Research Centre, University College London, London, UK

**Keywords:** Genetics, Molecular biology, Diseases, Medical research

## Abstract

To gain insight on dystrophin *(DMD)* gene transcription dynamics and spatial localization, we assayed the *DMD* mRNA amount and defined its compartmentalization in myoblasts, myotubes, and skeletal muscle biopsies of Duchenne muscular dystrophy (DMD) patients. Using droplet digital PCR, Real-time PCR, and RNAscope in situ hybridization, we showed that the *DMD* transcript amount is extremely reduced in both DMD patients’ cells and muscle biopsies and that mutation-related differences occur. We also found that, compared to controls, *DMD* transcript is dramatically reduced in the cytoplasm, as up to 90% of it is localized in nuclei, preferentially at the perinuclear region. Using RNA/protein colocalization experiments, we showed that about 40% of nuclear *DMD* mRNA is localized in the nucleoli in both control and DMD myogenic cells. Our results clearly show that mutant *DMD* mRNA quantity is strongly reduced in the patients’ myogenic cells and muscle biopsies. Furthermore, mutant *DMD* mRNA compartmentalization is spatially unbalanced due to a shift in its localization towards the nuclei. This abnormal transcript repartition contributes to the poor abundance and availability of the dystrophin messenger in cytoplasm. This novel finding also has important repercussions for RNA-targeted therapies.

## Introduction

Duchenne muscular dystrophy (DMD) is a severe X-linked rare disease (1 in 3500 to 5000 males born worldwide) due to pathogenic variations in the dystrophin (*DMD*) gene^1^. DMD pathogenesis is due to a cascade of events linked to the partial or complete lack of dystrophin protein (DYS) production in striated muscles (muscle and heart). Events include sarcolemma fragility, massive calcium influx, and cell necrosis, followed by apoptosis and cell death, thus triggering the entire cohort of inflammatory pathways, cell signaling alterations involving the nitric oxide synthase circuit, and failure of regeneration due to intrinsic satellite cell dysfunction^2^. The majority of *DMD* mutations are copy number variations (CNVs) like deletions or duplications. Less common types are small mutations and, very rarely, atypical mutations. Out-of-framing mutation types induce the complete lack of DYS in the severe Duchenne form, while in-frame mutations cause the milder allelic form, Becker muscular dystrophy (BMD), which maintains some DYS production, although with quantitative and qualitative abnormalities^1^.

In the last 15 years, very promising therapeutic approaches for DMD boys have been designed and carried out in several clinical trials^3,4^.

Although these approaches modulate nuclear pre-mRNA splicing (via exon skipping), mRNA translation (via codon reversion approach), or the expression of synthetic minigenes, which are concurrent with *DMD* gene transcription (via gene therapy), our knowledge of *DMD* transcript dynamics, cell compartmentalization, and spatial gene expression is very poor.

*DMD* gene transcription is a long and complex process which requires 16 h and is concurrent with splicing, which is finely regulated via several alternative splicing events including recursive splicing^5^. Adding further complexity to the *DMD* locus transcription, three full-length isoforms, driven by specific promoters (Dp427m, Dp427b, and Dp427p) and several 3’-end shorter isoforms (Dp260, Dp140, Dp116, Dp71) driven from internal promoters, are transcribed. All these isoforms are often tissue-specific, alternatively spliced and/or with different 3’-untranslated regions, and relevant for dystrophin function on their production sites^6–8^.

Novel RNA hybridization techniques with chromogenic or fluorescent RNAscope/BaseScope-based methods allow the design of highly specific RNA probes. RNAscope has recently been used to study *DMD* transcript dynamics in healthy wild-type (WT) and dystrophic (*mdx*) mouse muscle and in healthy and dystrophic canine embryos^9–11^.

These techniques showed that immature transcripts are very abundant while mature transcripts are short-lived, even in control murine muscle. In canine embryos, the shorter *DMD* isoforms seem to be associated with proliferation and migration, while the longer isoforms are associated with terminal lineage commitment. RNAscope was also applied in studying *DMD* transcript localization in dystrophin canine brain^12^.

A reduced local mRNA synthesis was shown both in *mdx* and in DMD cells^11^. Authors showed that *DMD* mRNA molecules naturally localize in the nuclear compartment and they explored if nonsense mediated decay (NMD) plays a role in reducing *DMD* mRNA synthesis. They demonstrated that premature termination codon occurrence affects *DMD* locus transcription dynamics and suggested a possible epigenetic mechanism as being implicated in mRNA reduction.

*DMD* mRNA cell compartmentalization and transcription dynamics are poorly understood processes. After completing the transcription in the nucleolus, the mRNAs typically dynamically associate with RNA binding proteins (RBPs) and, following transcript cleavage and polyA tail addition, are assembled in complex molecules called messenger ribonucleoproteins (mRNPs) which simply and passively diffuse into the nucleoplasm, reach the nuclear pores, and then translocate across them in a few seconds-long process. mRNA transport across nuclear membrane might be active (ATP-dependent) or passive, depending on the mRNA molecular weight. Normally, mRNA of up to 40 kDa diffuse out, while larger molecules (like *DMD*) need transporters such as nuclear transport receptors. Notably, export of mRNPs is closely related to quality-control mechanisms since spliced mRNAs are efficiently exported, while partially spliced intron-containing mRNAs usually accumulate in the nucleus^13^.

Many other factors orchestrate the transcript dynamic, including microtubules, which are crucial for the RNA distribution in skeletal muscle. Interestingly, it is well known that alteration in microtubule architecture occurs in *mdx* muscle^14,15^.

We assayed the *DMD* mRNA amount and its compartmentalization by droplet digital PCR (ddPCR), Real-time PCR, and two RNAscope probes in myoblasts, myotubes, and skeletal muscle biopsies from 10 DMD patients carrying different pathogenic variations and 2 WT.

We observed a global reduction of *DMD* transcript both in cells and in muscle tissues, which is more evident in patients carrying specific mutations. This reduction dramatically occurred in the cytoplasmatic compartment and resulted in a massive nucleus/cytoplasm transcript unbalancing with up to 90% of *DMD* transcript accumulated in nuclei. We also observed an enrichment of *DMD* transcript in the perinuclear region.

These findings are important to understand *DMD* transcript dynamics and cell compartmentalization and may have an impact on RNA-targeted therapies.

## Results

### Global reduction and nuclear/cytoplasmic unbalancing of *DMD* mRNA in nuclei and cytoplasm of DMD immortalized myoblasts

Using ddPCR, we quantified the Dp427m isoform in nuclear and cytoplasmic separated fractions of WT1 and 3 DMD patients’ (DMD2, DMD3, and DMD5) immortalized myoblasts (Table 1).

**Table 1:** List of enrolled subjects and biological material analyzed.

Sample name (ID)	Phenotype	Age	DMD mutation	Sample type
*WT1*	Healthy donor	25	/	Immortalized myoblasts and myotubes from semitendinosus muscle
*WT2*	Healthy donor	37	/	Tibialis anterior muscle
*DMD1*	DMD	4	del ex 42–43	Immortalized myoblasts from left quadriceps muscle
*DMD2*	DMD	2	del ex 44–50	Immortalized myoblasts and myotubes from left quadriceps muscle
*DMD3*	DMD	2	del ex 45–52	Immortalized myoblasts from left quadriceps muscle
*DMD4*	DMD	2	del ex 48–50	Immortalized myoblasts and myotubes from left quadriceps muscle
*DMD5*	DMD	10	del ex 52	Immortalized myoblasts and myotubes from tensor fasciae latae muscle
*DMD6*	DMD	5	c.1264G > T ex 11 p.(Glu422*)	Tibialis anterior muscle
*DMD7*	DMD	10	del ex 43	Tibialis anterior muscle
*DMD8*	DMD	8	del ex 45	Tibialis anterior muscle
*DMD9*	DMD	7	del ex 45	Tibialis anterior muscle
*DMD10*	DMD	9	del ex 45–50	Tibialis anterior muscle

The patients are listed based on the location of the mutation from 5′ to 3′ along the *DMD* gene.

**Figure 1:**
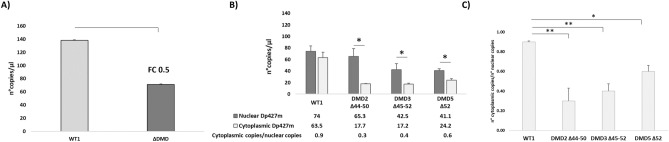
Evaluation of dystrophin transcript levels in WT1 and DMD immortalized myoblasts using ddPCR. The differences between WT and DMD cells were evaluated as fold change (FC). (**A**) The graph shows the reduction of total *DMD* transcript levels in DMD myoblasts compared to WT cells with FC of 0.5 (DMD copies/WT copies). ΔDMD represents the average of DMD2, DMD3, and DMD5. (**B**) The reduction of *DMD* transcript was especially due to a statistically significant low number of cytoplasmic copies compared to nuclear copies in all DMD immortalized myoblasts (cytoplasmic/nuclear ratio ranging from 0.3 to 0.6). (**C**) DMD cells showed a statistically significant cytoplasmic decrease of *DMD* transcript. The data are the mean of three separate experiments. Bar: SEM. **p < 0.001; *p < 0.05.

We observed a global reduction of Dp427m transcript in all DMD cells (Fig. 1A) with fold change (FC) values ranging from 0.4 to 0.6 (Fig. S1). DMD3 and DMD5 (carrying deletions which include exon 52), showed the lowest transcript amount (Fig. S1). When considering nuclear and cytoplasmic fractions, WT cells showed a balanced number of nuclear and cytoplasmic copies with a modest preferential nuclear localization, while DMD cells showed a statistically significant and dramatic cytoplasmic decrease of *DMD* transcript, almost entirely confined to the nuclei, with a cytoplasmic/nuclear ratio of 0.3 (DMD2), 0.4 (DMD3), and 0.6 (DMD5) (Fig. 1B). Although the extent of nuclear accumulation varies across patients, a statistically significant cytoplasmic depletion occurs in all of them (Fig. 1C).

**Figure 2:**
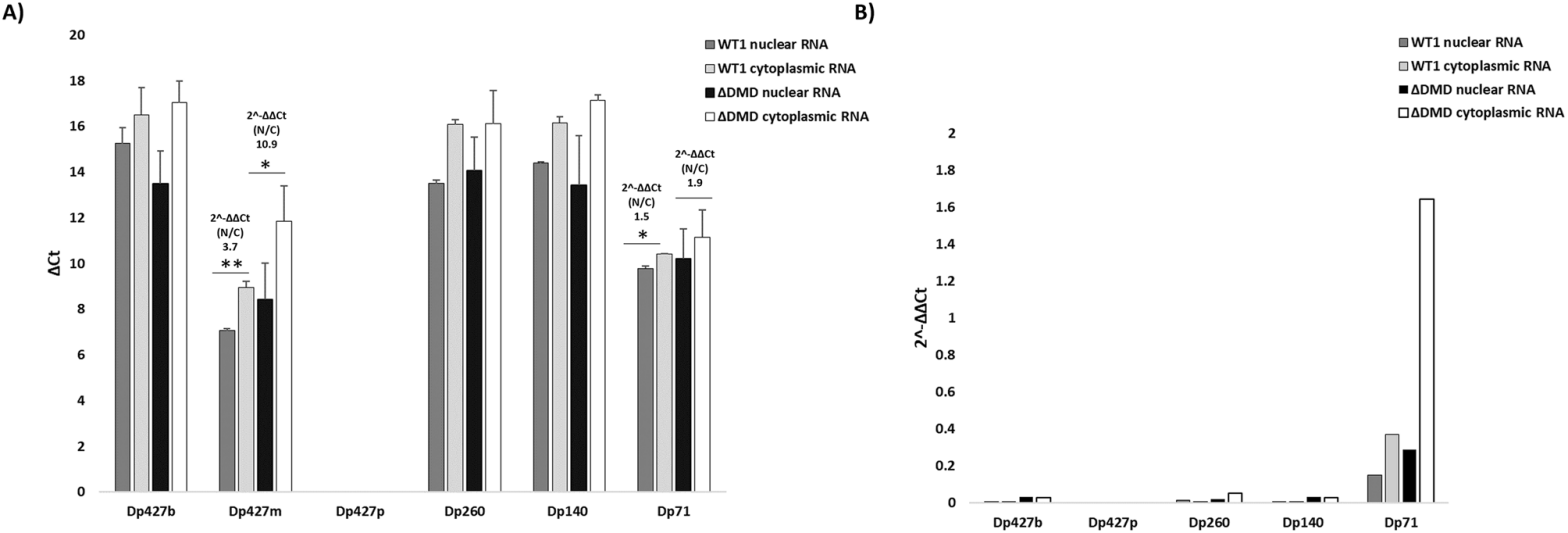
Expression profile of *DMD* isoforms in nuclear and cytoplasmic RNA from WT and DMD immortalized myoblasts using Real-time PCR. (**A**) Dp427m and Dp71 are the highest expressed *DMD* isoforms in myogenic cells. Low expression levels for Dp427b, Dp260, and Dp140 isoforms and the absence of expression of Dp427p isoform are observed. A statistically significant Dp427m nuclear/cytoplasmic unbalancing occurs mainly in DMD cells with 2^-∆∆Ct values of nuclear vs cytoplasmic fraction of 3.7 for WT1 and 10.9 for DMD. A similar Dp71 nuclear/cytoplasmic ratio is observed in WT and DMD with 2^-∆∆Ct values of nuclear vs cytoplasmic fraction of 1.5 and 1.9, respectively. Bar: SEM. ** *p* < 0.001; * *p* < 0.05. (**B**) 2^-∆∆Ct values of Dp427b, Dp427p, Dp260, and Dp140 isoforms (versus Dp427m) show that their expression can be considered negligible, while Dp71 is enriched in the cytoplasmic fraction of DMD cells.

To assess whether the transcript imbalance differently affects the *DMD* isoforms, we analyzed nuclear and cytoplasmatic fractions of WT1 and DMD (DMD2, DMD3, and DMD5) immortalized myoblasts by Real-time PCR. We showed that myoblasts are mostly enriched in Dp427m and Dp71 (Fig. 2A). Indeed, the 2^-∆∆Ct values of Dp427b, Dp427p, and Dp260 isoforms demonstrate that their expression is negligible (2^-∆∆Ct values ranging from 0.003 to 0.05, Fig. 2B). So, only Dp427m and Dp71 isoforms are represented in these cells. Real-time PCR results match ddPCR data and demonstrate a higher nuclear/cytoplasmic Dp427m unbalancing in DMD myogenic cells (2^-∆∆Ct value of 10.9, Fig. 2A) compared to WT cells (2^-∆∆Ct value of 3.7, Fig. 2A).

Less nuclear/cytoplasmic unbalancing is observed in Dp71 (2^-∆∆Ct values of 1.9, Fig. 2A).

It is to be noted that Dp71 (but not Dp427m) is enriched in the cytoplasmic fraction of DMD cells (Fig. 2B).

### RNAscope nuclear and cytoplasmatic localization and morphology in immortalized myoblasts and myotubes and muscle tissues

We performed RNAscope 2.5-Red assay in 5 DMD immortalized myoblasts (patients DMD1, DMD2, DMD3, DMD4, and DMD5), in 3 DMD myotubes (patients DMD2, DMD4, and DMD5), and in 6 DMD skeletal muscle biopsies (patients DMD6, DMD7, DMD8, DMD9, and DMD10). We also analyzed 2 control samples (Table 1).

The Polr2A (RNA Polymerase II Subunit A) probe was used as the positive control, while DapB (4-hydroxy-tetrahydrodipicolinate reductase) was used as the negative probe. RNAscope-Red assay visualization of either the positive or target probe is expected as a single red dot representing an individual mRNA molecule (Fig. S2, left panel). The negative control probe did not generate any signal (Fig. S2, right panel).

**Figure 3:**
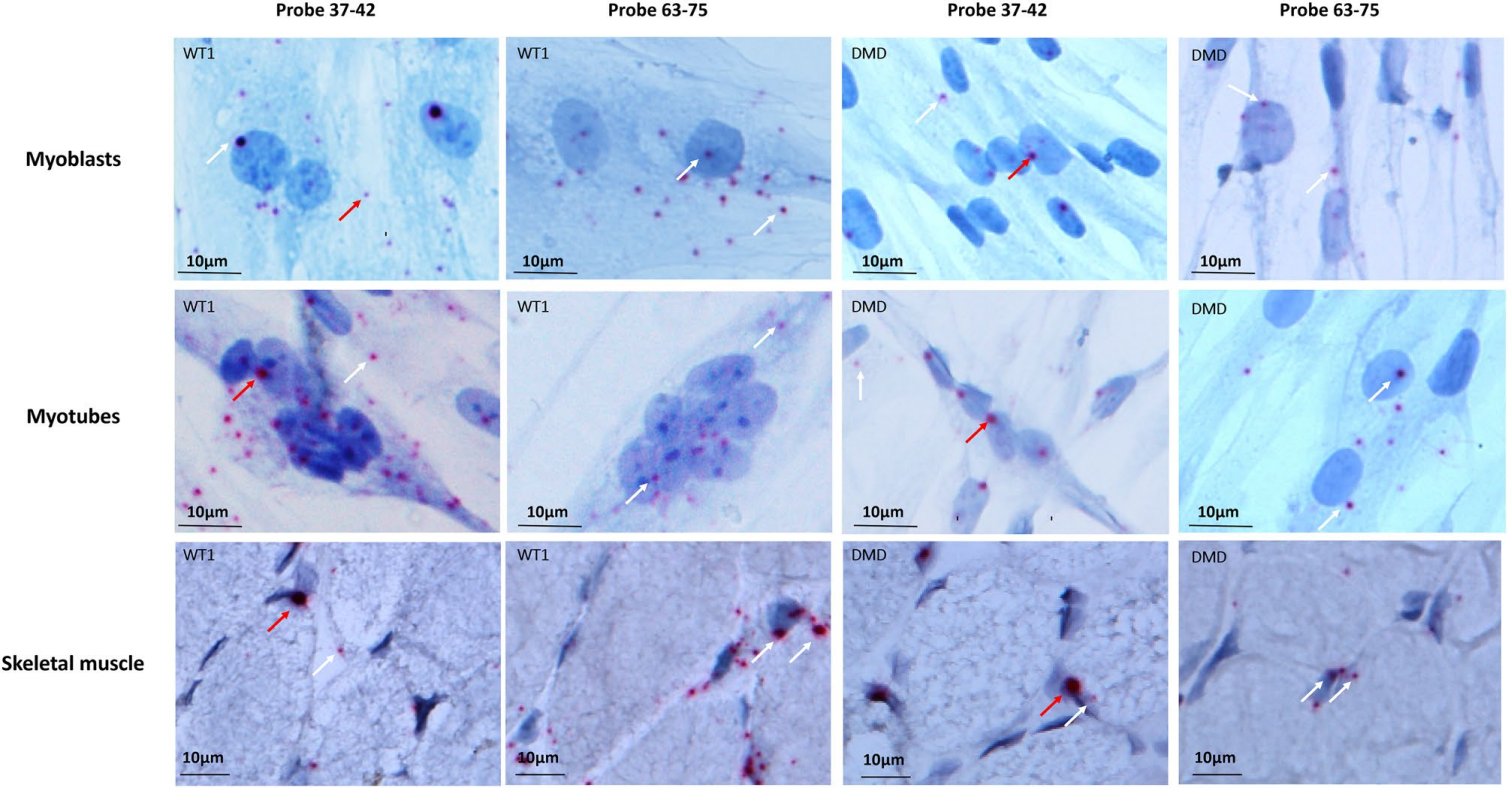
RNAscope in situ hybridization of immortalized myoblasts (upper panels), immortalized myotubes (medium panels), and skeletal muscle (SKM, lower panels) from WT and DMD individuals. A 37–42 ZZ probe targeting exons 37–42 and a 63–75 ZZ probe targeting exons 63–75 of the dystrophin transcript were used. Each single *DMD* transcript is represented as a distinct red dot. The 37–42 probe produces intense dot signals in both WT and DMD samples, mainly localized in nuclei (red arrows), while small dots are mainly localized in cytoplasm (white arrows). Probe 63–75 shows smaller dots (white arrows) in both nuclei and cytoplasm of WT and DMD. Counterstain: Gill’s Hematoxylin.

We used two RNAscope probes targeting exons 37–42 and exons 63–75, respectively, of the *DMD* gene. The 37–42 probe is expected to pick up Dp427m, Dp427b, Dp427p, and Dp260 and the 63–75 probe is expected to identify all isoforms, i.e. Dp427m, Dp427b, Dp427p, Dp260, Dp140, and Dp71. Based on our Real-time PCR data, which have profiled all *DMD* isoforms in the very same cells (Fig. 2), we were able to infer that RNAscope dots obtained with probe 37–42 represent Dp427m while those of probe 63–75 represent both Dp427m and Dp71. Figure 3 shows details of RNAscope in immortalized myoblasts, myotubes, and skeletal muscle from WT and DMD patients. The 37–42 probe (Dp427m isoform) generates intense dot signals, mainly localized in nuclei and perinuclear membrane, while small dots were mainly localized in cytoplasm; the 63–75 probe (Dp427m and Dp71 isoforms) showed smaller dots both in nuclei and cytoplasm. It is known that larger RNAscope dots correlate with nascent, not completely spliced, pre-mRNAs, while smaller dots represent fully spliced mRNA^9,10^.

Also, mRNA conformation changes due to RNP complexes concur in causing these different RNAscope signal intensities^16^. Thus, we conclude that differences in dot intensity are likely due to the different conformation of long, nuclear mRNA (as the full length Dp427m) which can be either partially spliced or enriched in RNPs. Conversely, cytoplasmic transcripts, which are fully spliced, or shorter mRNAs (as Dp71), generate smaller dots.

Sporadically and similarly to what has already been described in mouse models, some myoblast nuclei did not show any RNAscope signal^11^.

**Figure 4:**
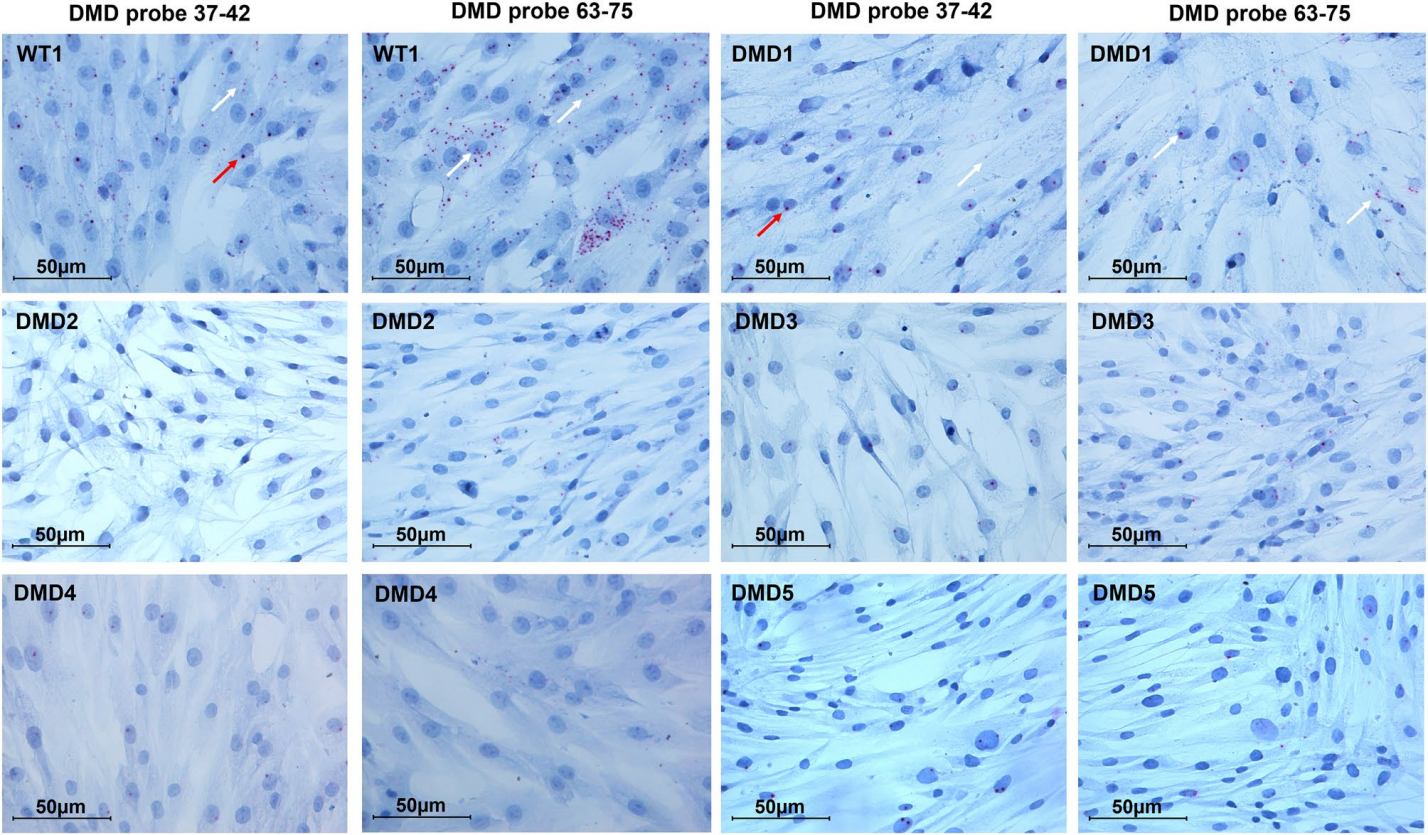
RNAscope in situ hybridization profile in immortalized myoblasts from WT and DMD individuals. Red arrows: intense and large dot signals. White arrows: small dots. Counterstain: Gill’s Hematoxylin.

DMD immortalized cells showed a marked decrease of dystrophin transcript signals (Fig. 4). Using the 63–75 probe, the number of dots was higher compared to the 37–42 probe, a fact that was quite expected since the 63–75 probe detects not only Dp427m but also the Dp71 isoform, the transcription of which is preserved in all studied DMD patients.

**Figure 5:**
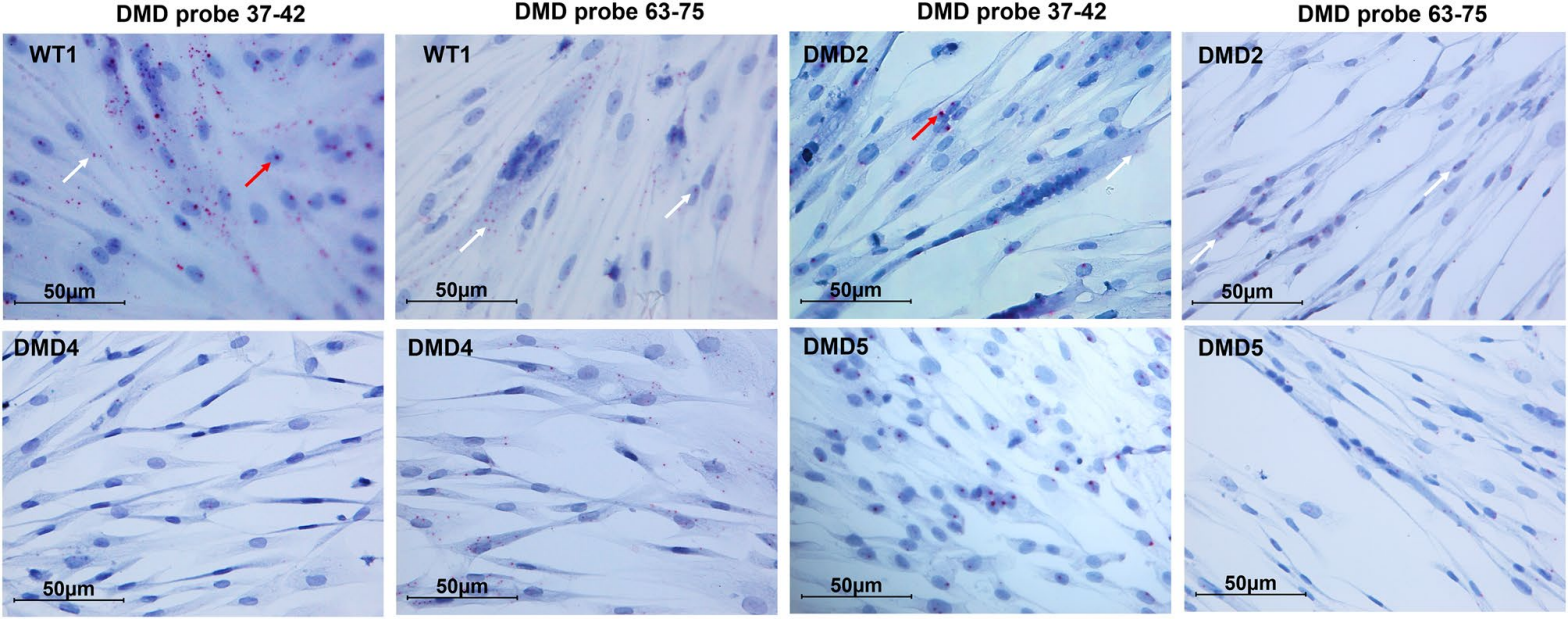
RNAscope in situ hybridization profile in immortalized myotubes from WT and DMD individuals. Red arrows: intense and large dot signals. White arrows: small dots. Counterstain: Gill’s Hematoxylin.

We have also performed RNAscope in immortalized myotubes obtained by differentiating WT1, DMD2, DMD3, and DMD4 immortalized myoblasts. Results fully recapitulate those seen in myoblasts in terms of dot intensity and transcript distribution (Fig. 5).

**Figure 6:**
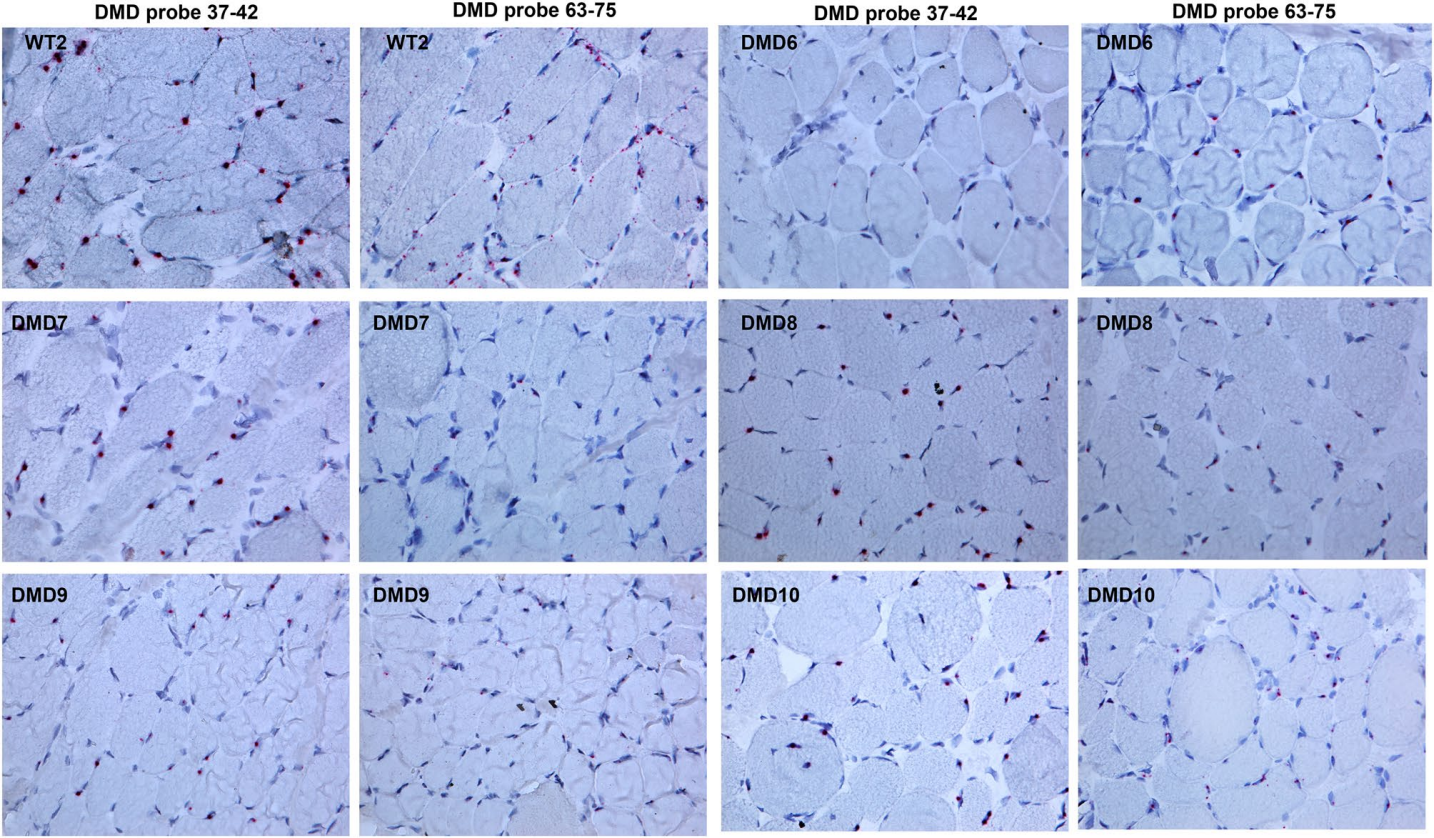
RNAscope in situ hybridization profile in skeletal muscle (SKM) from WT and DMD individuals. Counterstain: Gill’s Hematoxylin.

To explore the transcript distribution and morphology as well as in muscle tissue, we performed RNAscope in 5 DMD skeletal muscle biopsies (patients DMD6, DMD7, DMD8, DMD9, and DMD10) and in one control muscle biopsy (WT2) (Fig. 6). The morphology of the dots was the same as the one we demonstrated in myogenic cells. The 37–42 probe produces large dots in nuclei, while the 63–75 probe shows smaller dots, suggesting that this morphology is consistent with our explanation of a *DMD* mRNA-specific configuration in the cytoplasm and nucleus.

Some nuclei in both WT and DMD myotubes (Fig. 5) and muscle (Fig. 6) did not show any signals as seen in myogenic cells (Fig. 4).

### RNAscope signals semi-quantification in immortalized myoblasts and myotubes and in muscle tissues

We quantified total dystrophin transcript by counting the number of dots for each image and established the ratio between the number of dots and the number of nuclei.

**Figure 7:**
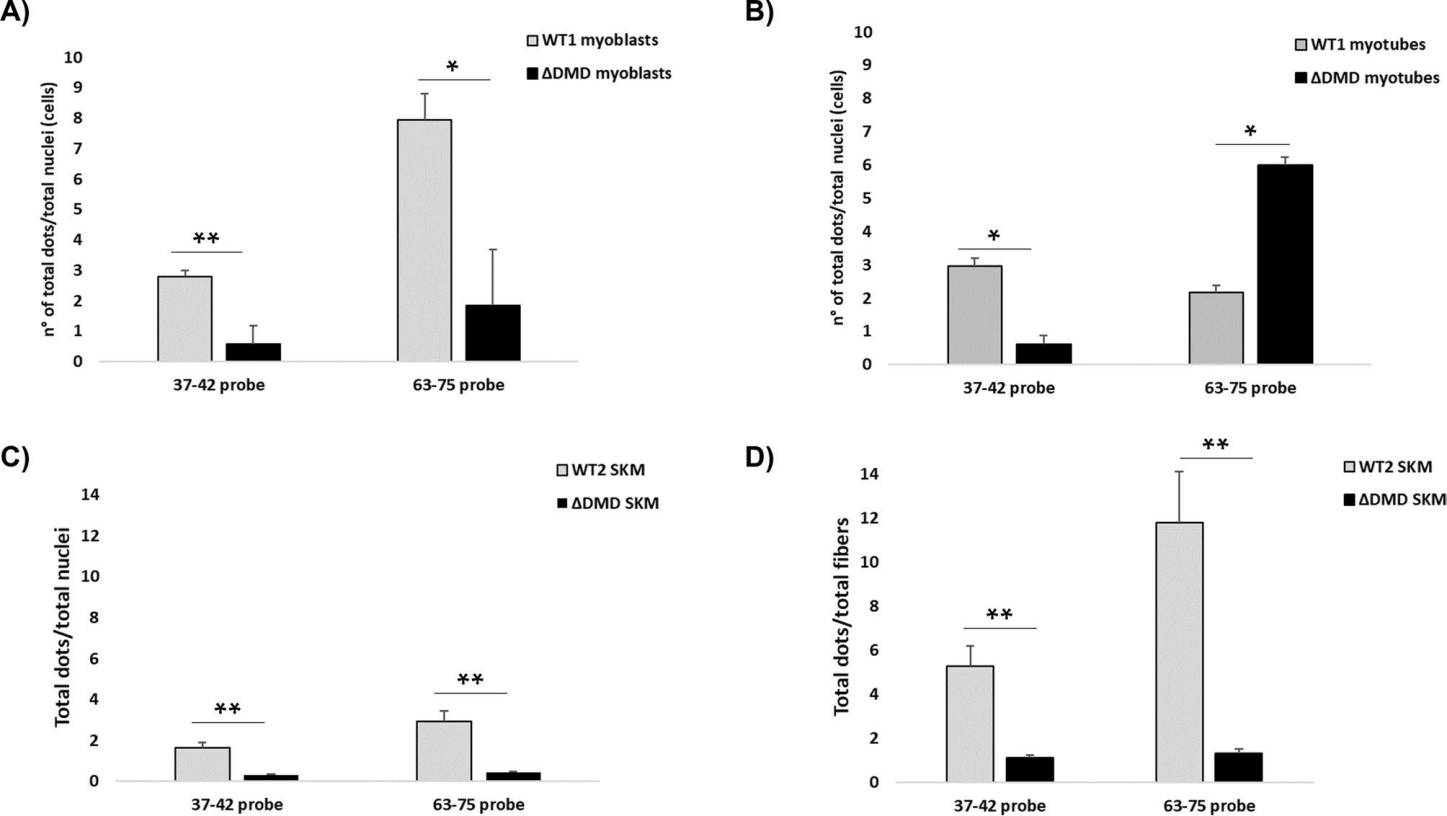
Comparison of *DMD* transcript levels between WT and DMD patients. (**A**) The quantification of total red dots per cell shows that the DMD immortalized myoblasts have significantly lower dystrophin transcript levels compared to WT cells using both the 37–42 probe (targeting full-length *DMD* isoforms) and the 63–75 probe (targeting both full-length and Dp71 isoforms). ΔDMD represents the average of DMD1, DMD2, DMD3, DMD4, and DMD5. (**B**) Using the 37–42 probe, DMD myotubes show lower full-length dystrophin transcript levels compared to WT1 cells. When using the 63–75 probe, we observed a high number of signals in DMD myotubes, possibly due to a different myogenic differentiation capacity between WT and DMD cells and, therefore, a presence of proliferating cells still expressing Dp71 isoform in DMD cultures. ΔDMD represents the average of DMD2, DMD4, and DMD5. (**C** and **D**) The quantification of total red dots per cell (**C**) and per fiber (**D**) shows that DMD muscles have lower dystrophin transcript levels compared to WT muscle using both the 37–42 and 63–75 probes. ΔDMD represents the average of DMD6, DMD7, DMD8, DMD9, and DMD10. Bar: SEM. **p < 0.001; *p < 0.05.

Using the 37–42 and 63–75 probes, *DMD* transcript amount is significantly reduced in DMD myoblasts (Fig. 7A).

We observed in patients’ myoblasts some mutation-dependent differences in *DMD* transcript amount using both probes (Fig. S3A). Indeed, DMD1 (∆42–43) was the patient with the highest mRNA amount, DMD2 and DMD4 (∆ including exon 50) showed intermediate levels, and DMD3 and DMD5 (∆ including exon 52) showed the lowest amount of transcript. It is to be noted that the cells of patient DMD1 also showed the highest Dp71 amount.

RNAscope dot quantification in myotubes showed that DMD samples invariably have a lower Dp427m amount (probe 37–42) compared to the WT (Fig. 7B). As seen in patients’ myoblasts, DMD5’s myotubes (∆52) showed the lowest amount of transcript (Fig. S3B). Using the 63–75 probe targeting both Dp427 and Dp71, we observed a higher number of dots in patients DMD2 and DMD4 (Fig. S3B). This is very likely due to the presence of Dp71, since myotubes might not be fully differentiated and may still be expressing some residual Dp71 isoform^17,18^.

Quantification of RNAscope in muscle is reported in Fig. 7C, D and fully recapitulates results in cells. A very significant reduction of *DMD* transcript occurred in all muscle biopsies using both probes.

Data on expression of Dp71 in skeletal muscle are controversial since some studies found Dp71 absent in mature muscle fibers^17^, while others demonstrated the presence of Dp71 either in DMD patients’ muscle^18^ and in human control skeletal muscle^19^ by using highly sensitive methods. These data suggest that Dp71 production in DMD muscle might be a secondary event related to muscle degeneration and regeneration.

We performed Real-time PCR on total mRNA from WT2 and DMD (DMD6, DMD7, DMD8, DMD9, and DMD10) skeletal muscles to assay *DMD* isoforms. We found that Dp427m (main isoform expressed, Fig. S4A) and Dp71 are expressed in all samples, overlapping the results of the immortalized myoblasts.

Nevertheless, since the Dp71 expression is low in WT and DMD muscles, we conclude that the two RNAscope probes have identified mostly Dp427m.

Note that, unlike cells, no mutation-related differences in *DMD* transcript abundance were observed in DMD skeletal muscle (Fig. S3C and D).

### Nuclear/cytoplasm *DMD* transcript unbalancing in immortalized myoblasts and myotubes and muscle tissues

We evaluated the percentage of DMD RNAscope dots in the nucleus and cytoplasm, counted as nuclear or cytoplasmic dots/total dots in cells and muscles from WT and DMD patients.

**Figure 8:**
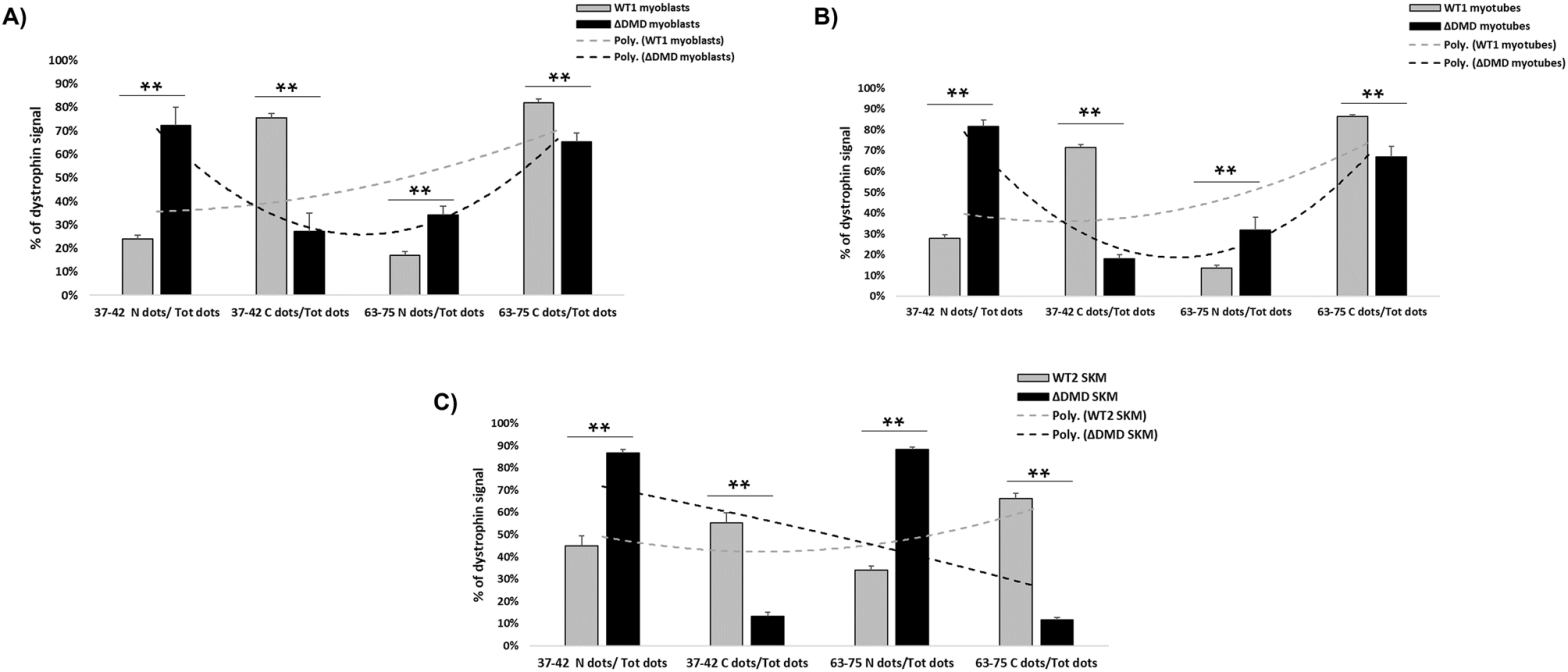
RNAscope ISH in immortalized cells and skeletal muscles from WT and DMD patients. The % of dystrophin signal in nuclear or cytoplasmic compartments was calculated as a ratio of total Nuclear (N) or Cytoplasmic (C) dots and Total dots (Tot). Using the 37–42 probe, we showed that *DMD* mRNA is mostly localized into nuclei of DMD immortalized myoblasts (**A**), myotubes (**B**), and skeletal muscle (SKM, **C**). WT showed higher levels of *DMD* RNA into cytoplasmic fraction using both the 37–42 and 63–75 probes. The 63–75 probe showed an opposite trend in DMD myoblasts and myotubes in which a higher number of dystrophin signal is observed in the cytoplasmic compartment compared to the 37–42 probe, due to the additional detection of Dp71 isoform (**A** and **B**). When using the 63–75 probe, we did not observe an increased number of cytoplasmic dots in DMD skeletal muscle (**C**). The difference in *DMD* nuclear and cytoplasmic localization between WT and DMD samples are statistically significant for all cells and muscles. The different profile of *DMD* transcript localization is highlighted by the polynomial curves (dotted curve). Poly. Polynomial curve. ΔDMD myoblasts represent the average of DMD1, DMD2, DMD3, DMD4, and DMD5. ΔDMD myotubes represent the average of DMD2, DMD4, and DMD5. ΔDMD SKM represent the average of DMD6, DMD7, DMD8, DMD9, and DMD10. Bar: SEM. **p < 0.001.

We summarized in Fig. 8 the averages of the data from DMD patients (ΔDMD) and we observed a range of 72–87% of the 37–42 probe localized into DMD nuclei in all sample types, myoblasts, myotubes, and skeletal muscles, while WT1 and WT2 showed a clear predominant cytoplasm localization (range 55–76%). The differences between DMD nuclear and cytoplasmic localization between WT and DMD are statistically significant in all DMD samples. Interestingly, the 63–75 probe mainly localized in cytoplasm in DMD cells (65–67%, Fig. 8A and B), while 84–93% of the 63–75 dots localized in nuclei in DMD muscle (Fig. 8C).

**Figure 9:**
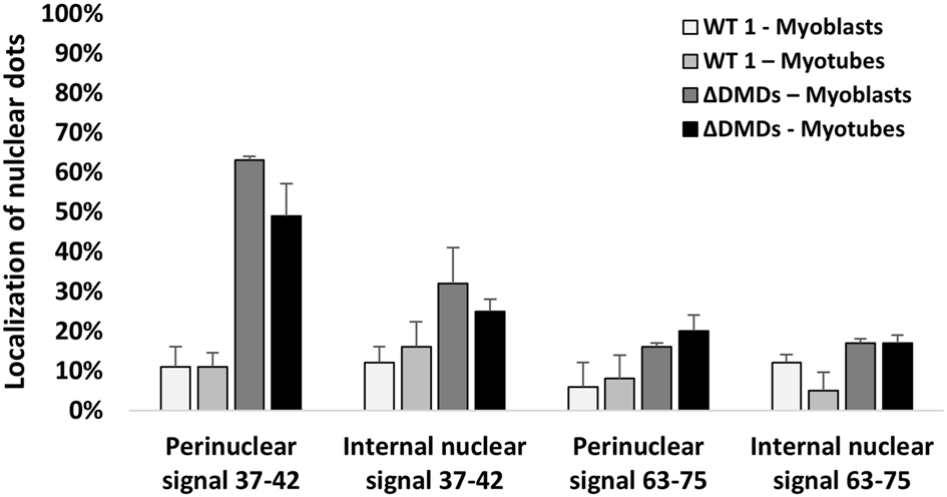
Percentages of RNAscope dots in perinuclear and internal nuclear region of immortalized myoblasts and myotubes from WT1 and DMD. The ΔDMD percentage is the average of five DMD patients’ myoblasts (DMD1, DMD2, DMD3, DMD4 and DMD5) and three DMD patients’ myotubes (DMD2, DMD4 and DMD5). The figure shows that most of the nuclear *DMD* mRNA detected by the 37–42 probe localize in the perinuclear region of DMD cells. Using the 63–75 probe, the % of perinuclear signals decrease in both DMD myoblasts and myotubes. Bar: Standard Deviation.

This different trend is likely due to less enrichment of skeletal muscle in Dp71, a fact that allows us to conclude that almost all *DMD* transcript in nuclei of DMD muscles consists of Dp427m (Fig. 8). Figures S5–S7 show the DMD nuclear and cytoplasmic profile of myoblasts (Fig. S5), myotubes (Fig. S6), and skeletal muscle (Fig. S7) from each DMD patient. Mutation types do not influence the N/C ratio in cells and skeletal muscle. We also calculated the percentage of *DMD* RNA signals localized in the perinuclear region of cells, and we found a marked enrichment of perinuclear 37–42 probe dots in DMD cells, which accounts for 49–63% of transcripts, while only 16–20% of 63–75 probe dots is perinuclearly localized (Fig. 9). This result suggests that the enrichment of RNA at the perinuclear regions of DMD myogenic cells is mainly due to the mutated Dp427m, thus supporting our findings.

### Nuclear dystrophin signals are enriched at nucleoli of immortalized myoblasts

**Figure 10:**
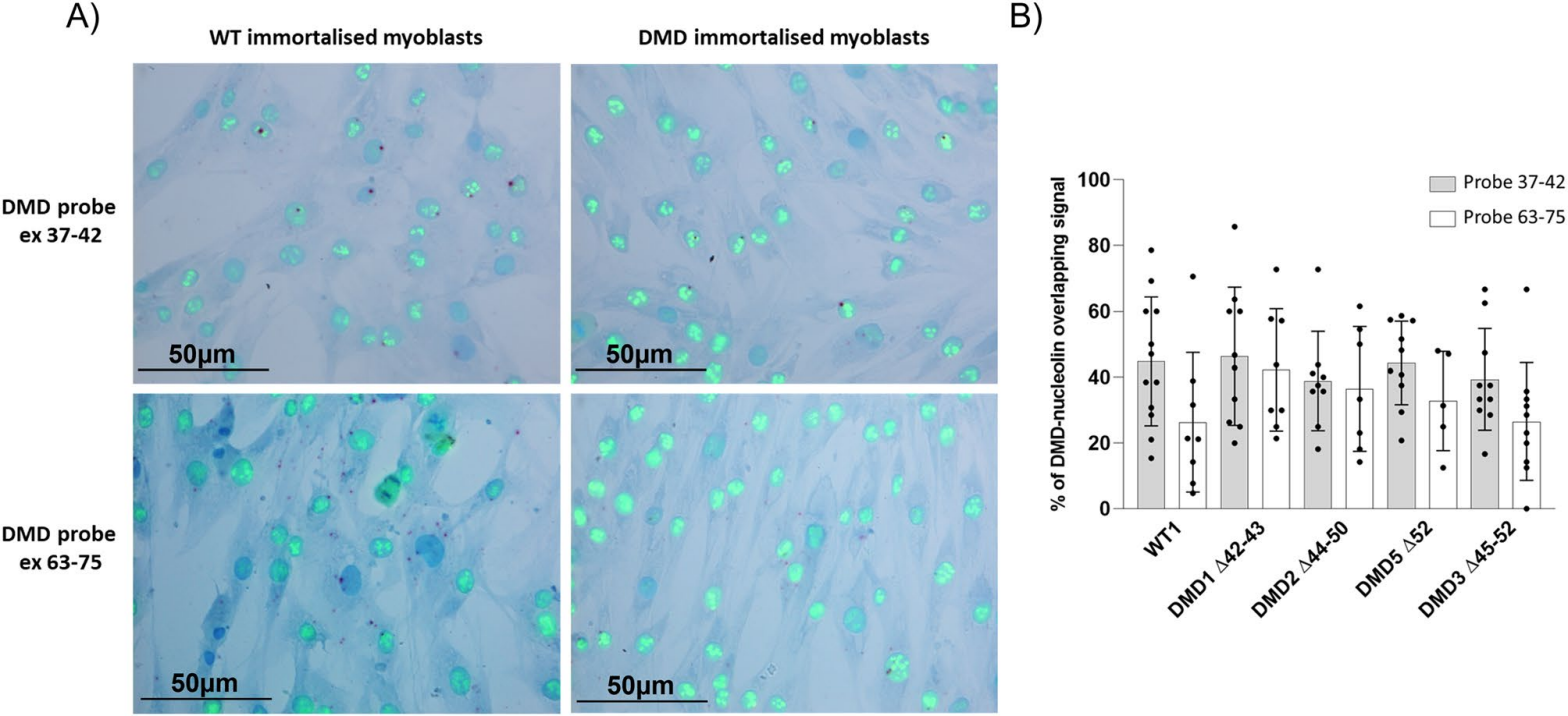
Dual RNAscope and immunostaining in WT1 and DMD immortalized myoblasts. (**A**) 37–42 and 63–75 RNAscope probes were used in combination with a monoclonal antibody against the human nucleolin protein. Each single *DMD* transcript is represented as a distinct red dot, whereas nucleolin protein is represented as a green signal. Counterstain: Gill’s Hematoxylin. (**B**) Colocalization of dystrophin transcript and nucleolin protein in immortalized myoblasts. Using the 37–42 probe, we found about 45% in WT1 and a mean of 42% in DMD of Dp427 overlapping with nucleolin signals. No statistically significant differences were observed between samples. Using probe 63–75, we showed lower dystrophin and nucleolin colocalization in both WT1 (26%) and DMD (mean of 34%). No statistically significant differences were found for 63–75 signal between WT1 and DMD. Bar: SEM. **p < 0.001; *p < 0.05.

Nucleoli participate in mRNA by exporting and retaining altered mRNAs, thus playing an important role in mRNA trafficking, decay surveillance, and export^20,21^. To better explore the relationship between the dramatic cytoplasm reduction of Dp427m we found in all DMD sample types, we assayed by duplex RNAscope/immunocytochemistry (ICC) control (WT1) and DMD myoblasts (patients DMD1, DMD2, DMD3, and DMD5) using the 37–42 or 63–75 probes and nucleolin antibody (Fig. 10).

Morphologically, both control WT and DMD myoblasts showed a vast majority of nuclei in interphase where nucleolin is predominantly localized at the outer layer of the nucleoli (Fig. 10A).

Using the 37–42 probe (Dp427m), we found that in DMD samples, 42–45% of Dp427 mRNA colocalized with nucleolin signals, whereas using the 63–75 probe (Dp427m + Dp71), 34% of *DMD* mRNA colocalized with nucleolin (Fig. 10B). In WT samples, only 26% of probes colocalized with nucleolin (Fig. 10B). This finding clearly shows that the majority of Dp427m does associate with nucleoli while Dp71 does not, since it is mainly cytoplasmic. Again, this supports our RNAscope data.

Furthermore, we performed a coimmunoprecipitation of total RNAs with nucleolin protein in WT1 and DMD myoblasts (DMD1, DMD2, DMD3, and DMD5). Real-time PCR of *DMD* transcripts in all immunoprecipitated RNAs in both WT1 and DMD samples did not identify any specific *DMD* isoforms (Fig. S8), suggesting that *DMD* mRNA colocalizes to, but not directly binds, nucleoli.

We also evaluated the size of nuclei and the number of nucleoli, and we did not find variations between WT1 and DMD cells where nuclei diameter average (67 and 63 µm, respectively) and number of nucleoli per cell (3 nucleoli/cell) are very similar (Table 2).

**Table 2:** Evaluation of nuclei size and number of nucleoli in WT1 and DMD immortalized myoblasts.

Sample name (ID)	Total analyzed nuclei	Nuclei diameter mean (µm)	Mean nucleoli/cell
*WT1*	105	67	3
*DMD1 ∆42–43*	75	65	3
*DMD2 ∆44–50*	136	64	3
*DMD3 ∆45–52*	110	60	3
*DMD5 ∆52*	141	64	4

The table shows that WT and DMD cells do not present variations in nuclei diameter average and number of nucleoli per cell.

ΔDMD myoblasts represents the average of DMD1, DMD2, DMD3, DMD4, and DMD5.

## Discussion

We studied the dystrophin transcript amount, localization, and spatial distribution in human cultured myogenic cells and in muscle biopsies from controls (WT) and 10 DMD patients.

Using ddPCR, Real-time PCR, and RNAscope, we confirmed a global *DMD* transcript reduction occurring in DMD samples, cells, and muscle tissue, corroborating previously reported findings^11,22^.

In addition, using Real-time PCR, we observed that this reduction is mainly due to low Dp427m levels.

RNAscope provides both semi-quantitative results with single molecule detection at single-cell resolution and cell-specific expression information in a morphological context. Thus, our RNAscope results allowed us to add clues about *DMD* transcript spatial localization by defining its distribution within specific sub-regions/compartments of myogenic cells and muscle tissue.

As a completely novel observation, we demonstrated that the *DMD* transcript reduction we observed in patients is mainly caused by a dramatic decrease of *DMD* mRNA in cytoplasm, as we observed both in cells and in skeletal muscle.

Interestingly, we found differences in DMD cells depending on mutations. DMD patients’ cells carrying a deletion which includes exon 52 showed a very reduced *DMD* transcript, possibly related to the role of intron 52 regions in the chromatin shape which regulates *DMD* transcription^23^. Indeed, we previously reported a unique pausing site of RNA pol II in intron 52, which is almost invariably the site of breakpoint in exon 52 deletions. RNA pol II can enter a paused or stalled status immediately downstream of the transcription start site before productive elongation occurs. Therefore, we suggested that this region is crucial for dystrophin transcriptional dynamics^23^.

In the context of mutation-specific effects, we noted that DMD patients carrying a deletion of exons 42–43 showed the highest Dp71 levels at RNAscope ISH. By Real-time PCR assay, we also observed enrichment in Dp71 in the cytoplasm of DMD myoblasts.

Interestingly, Dp71 overexpression was already described in a del52 mice model^24^ as well as in DMD skeletal muscle, where it plays a possible role in myoblast cell proliferation and satellite cell activation^18^.

We also reported that WT nuclear fraction shows a moderate enrichment of Dp427m, a finding fully in line with previous published data^9,11^. Additionally, we found that not all nuclei of human muscle cells/tissue express dystrophin transcripts. An explanation could be that not all muscle loci are transcriptionally active at a given time as previously proposed for some muscle genes with a stochastic expression in mouse skeletal muscle^25^.

Through RNAscope localization studies, we demonstrated an impressive, unbalanced nuclei/cytoplasm *DMD* mRNA repartition in all DMD myoblasts, myotubes, and muscle biopsies with a predominant transcript nuclear localization (up to 90% of mRNAs) with a very poor mRNA representation in cytoplasm. WT myogenic cells and muscles show a rather balanced transcript distribution with about 60% of *DMD* mRNA in cytoplasm.

The large and intense signals in nuclei shown by the 37–42 probe are consistent with the occurrence of intermolecular RNA–RNA interactions that contribute to RNP granule formation, which are biomolecular condensates. These nuclear RNP granules include histone locus bodies and paraspeckles, which are known to participate at transcription start sites^16^. Thus, we suggest that the intense dots we observed using the 37–42 probe, and mostly identifying Dp427m, reflect a more complex transcriptional stage involving this *DMD* region and an interesting fact that would deserve further studies.

Our novel finding concerns the *DMD* mRNAs localization in the nucleolar compartment of human samples, which recapitulates what was once reported in rat myoblasts for several mRNA^26^. We demonstrated that the Dp427 is preferentially associated with nucleoli, indicating that the large *DMD* mRNA is mostly processed within the nucleolar compartment.

Our data suggest that nucleoli may act as a checkpoint for *DMD* transcript quality and quantity assessment before it is exported into cytoplasm, affecting the trafficking and exportation of mutated *DMD* mRNAs^20,21^.

In conclusion, our findings unequivocally show that mutated *DMD* mRNA is globally reduced in DMD cells and muscle tissues, and that this reduction is due to a dramatic decrease of the *DMD* messenger in the cytoplasmatic fraction, which is likely due to mutated *DMD* transcript nuclear localization. This leads to a consequent unbalanced spatial distribution with poor or absent mRNA availability in cytoplasm for protein translation. This unbalanced compartmentalization occurs for about 100% of Dp427m transcript, which remains confined at the perinuclear membrane.

We also suggest that nucleoli may act as a checkpoint for *DMD* transcript quality and quantity assessment by playing a role in its nuclear accumulation.

Interestingly, *DMD* transcript reduction appears to be mutation-unique behaviors that will require further assessment with a wider range of mutations.

We believe that our scientific contribution provides important clues to understanding *DMD* transcript dynamic and spatial distribution in order to design RNA targeted therapies.

## Materials and methods

All methods were carried out in accordance with relevant guidelines and regulations.

### Approval statement

All experimental protocols were approved by the University Hospital & University of Ferrara (AOUF).

Ethical Approvals: Number 9/2005; Number 841/2020/Sper/AOUF.

Informed consent was obtained from all subjects enrolled in the study.

### Enrolled subjects

The enrolled individuals are listed in Table 1. Immortalized myoblasts from 5 DMD patients and one healthy donor (WT1) were obtained from the MRC Centre for Neuromuscular Disorders Biobank London. Cells were cultured in high-glucose DMEM (GIBCO), supplemented with 20% fetal bovine serum (FBS; GIBCO) and antibiotic/antimycotic solution (Sigma). Myoblasts were induced to differentiate to myotubes after plating on Matrigel (Corning) coated four-well chamber slides in differentiation medium (DMEM with 2% FBS) for 5 days.

Muscle biopsies were collected from 5 DMD patients and one control subject (WT2) after informed consent for research purposes.

### Isolation of RNA from cells and muscle

The cytoplasmic and nuclear RNA purification Kit (Norgen, USA) was used to isolate and purify cytoplasmic and nuclear RNA from myoblasts and myotubes following manufacturer’s protocol.

Total RNA was isolated from WT2 and DMD patients’ skeletal muscles using the RNeasy-kit (QIAGEN, Chatsworth, CA, USA) according to the manufacturer’s instructions.

RNA was reverse transcribed into cDNA using random primers and the High-Capacity cDNA Reverse Transcription Kit (Applied Biosystems). RT-PCR was performed on β-actin to verify cDNA synthesis.

### Real-time PCR on WT immortalized myoblasts and skeletal muscle

Real-time PCR was used to quantify the full-length and short *DMD* isoforms in both nuclear and cytoplasmic fractions of immortalized myoblasts from WT1 and DMD2, DMD3, and DMD5. In addition, Real-time PCR was also performed on total RNA from WT2 and all DMD skeletal muscles (DMD6, DMD7, DMD8, DMD9, and DMD10).

Custom Taqman assays (Thermo Fisher Scientific) were designed to target the unique region of full-length dystrophin isoforms (Dp427b, Dp427m, and Dp427p) and short isoforms (Dp260, Dp140, and Dp71). Primer pairs and probe sequences are listed in Supplementary Table 1.

3 ng of cDNA of each sample were used for Real-time PCR. ß-actin was used as a housekeeping reference gene. Data were analyzed according to the comparative Ct method^27^. For statistical analysis, all data were analyzed by means of Student’s t-test. The data results are based on the average of three separate experiments. Bar: SEM. **p < 0.001; *p < 0.05.

### *DMD* mRNA analysis by droplet digital PCR (ddPCR) in immortalized myoblasts

The expression of Dp427m in both nuclear and cytoplasmic fractions of immortalized myoblasts from WT1 and DMD (DMD2, DMD3, and DMD5) was performed using ddPCR technique by the QX200ddPCR system (Bio-Rad, Hercules, CA USA)^28^. Custom Taqman assay (Thermo Fisher Scientific) were designed to target the unique region of the Dp427m (Table S1).

3 ng of cDNA of each sample were used for ddPCR.

Data analysis was carried out using Quanta Soft software (Bio-Rad, Hercules, CA USA). The final Dp427m concentration was reported as copies/µl.

For statistical analysis, all data were analyzed by means of Student’s t-test. Data results are based on the average of three separate experiments. Bar: SEM. ** *p* < 0.001; * *p* < 0.05.

### RNAscope and dual RNAscope/immunocitochemistry (ICC) assays

In situ hybridization (ISH) was performed using RNAscope 2.5 HD Reagent assay Red (ACD BIO). Polr2A (RNA Polymerase II Subunit A) was used as the positive control probe, while DapB (4-hydroxy-tetrahydrodipicolinate reductase) was used as the negative probe.

Two independent inventoried probes targeting *DMD* transcripts were used:

- Probe Hs-DMD-Dp427m-E37-E42 contains 15 Z pairs and targets the nucleotides between 5400 and 6361 of NM_004006 (corresponding to exons 37–42 region).
- Probe Hs-DMD contains 20 Z pairs and targets the nucleotides between 9511 and 10,902 of NM_004006 (corresponding to exons 63–75 region).

RNAscope was performed according to the manufacturer’s instructions with the following optimizations: incubation with 10% Neutral Buffered Formalin (NBF) for 60 min and incubation with Amp 6 for 60 min.

A dual ISH/ICC technique was used to visualize and colocalize the dystrophin signal with the nucleolin protein. ICC was performed after the ISH protocol using the anti-nucleolin C23 MS-3 monoclonal antibody diluted 1:100 (Santa Cruz) and followed by anti-mouse FITC-conjugated secondary antibody.

All images were observed with a Nikon Eclipse 80i fluorescence microscope (Nikon Instruments) connected to a high-resolution CCD camera (Nikon Instruments) at 40 × magnification.

### RNAscope image analysis and calculations

A semi-quantitative assessment of RNAscope was performed by counting:

- The total number of red dots per image (each dot represents a single dystrophin RNA molecule);
- The total number of nuclei per image (indicating the number of analyzed cells);
- The total number of red dots localized inside, in the perinuclear, and outside the nuclei per image (indicating the sub-cellular localization of dystrophin RNA molecules in nuclear, perinuclear, and cytoplasmic compartments).

Ten different images were analyzed for each sample and a total number of cells ranging from 400 to 2200 were evaluated for each subject.

The global dystrophin transcript quantification was calculated as total number of red dots/total number of nuclei (or total number of fibers for the SKM).

The subcellular localization of dystrophin transcript in the cytoplasmic compartment was calculated as total number of cytoplasmic red dots/total number of red dots × 100.

The subcellular localization of dystrophin transcript in the perinuclear region was calculated as total number of perinuclear red dots/total number of red dots × 100.

The subcellular localization of dystrophin transcript in the internal nuclear region was calculated as total number of internal nuclear red dots/total number of red dots × 100.

The subcellular localization of dystrophin transcript in nuclei (internal nuclear region + perinuclear region) was calculated as total number of nuclear and perinuclear red dots/total number of red dots × 100.

The colocalization of dystrophin transcript with nucleolin protein was evaluated by counting the number of red dots associated with the nucleolin green signal/total number of red dots × 100.

For statistical analysis, all data were analyzed by means of Student’s t-test. Data results are based on the average of ten different images. Bar: SEM. **p < 0.001; *p < 0.05.

### RNA-binding protein immunoprecipitation (Magna RIP)

Approximately 1 × 10^7^ myoblasts from WT1 and DMD (DMD1, DMD2, DMD3, and DMD5, Table 1) were harvested and lysed by resuspension in the Magna RIP (Millipore) lysis buffer. Nucleolin protein was immunoprecipitated with anti-nucleolin C23 MS-3 mouse monoclonal antibody (5 µg; Santa Cruz) using the Magna RIP RNA-Binding Protein Immunoprecipitation Kit (Millipore). Mouse IgG antibody was used as negative control of the immunoprecipitation reaction. Coimmunoprecipitated RNAs were isolated according to the manufacturer’s instructions. Assessment of RNA quality was analyzed on RNA nano chip using Agilent’s Bioanalyzer.

## Supplementary materials

### Real-time PCR on immunoprecipitated RNA by Magna RIP

RNA was reverse transcribed into cDNA using random primers and the High-Capacity cDNA Reverse Transcription Kit (Applied Biosystems).

Transcript quantification of Dp427m and Dp71 *DMD* isoforms was performed using custom TaqMan expression assays (Applied Biosystems, Table S1). RPL13A was used as positive control of nucleolin protein immunoprecipitation^29^. Data were analyzed according to the comparative Ct method.

### Supplementary Information

Supplementary Information.

## Data Availability

RNA-seq data for this study have been deposited in the GEO database under accession number GSE162108.
