# Supplementary material for: mRNA in situ hybridization exhibits unbalanced nuclear/cytoplasmic dystrophin transcript repartition in Duchenne myogenic cells and skeletal muscle biopsies
Source: Sci Rep. 2023 Sep 24;13:15942. doi: 10.1038/s41598-023-43134-6 (PMC10518324; doi:10.1038/s41598-023-43134-6)
Supplement: Supplementary file 1 — Supplementary Information. [file 41598_2023_43134_MOESM1_ESM.docx]

**Supplementary figure 1 (S1).**

The graph shows the reduction of total *DMD* transcript levels in all analyzed DMD myoblasts by ddPCR. The differences between WT and DMD cells were evaluated as fold change (FC, DMD copies/WT copies) with values ranging from 0.4 to 0.6 FC.

Bar, standard error (SEM); ** p< 0.001; * p< 0.05.

**
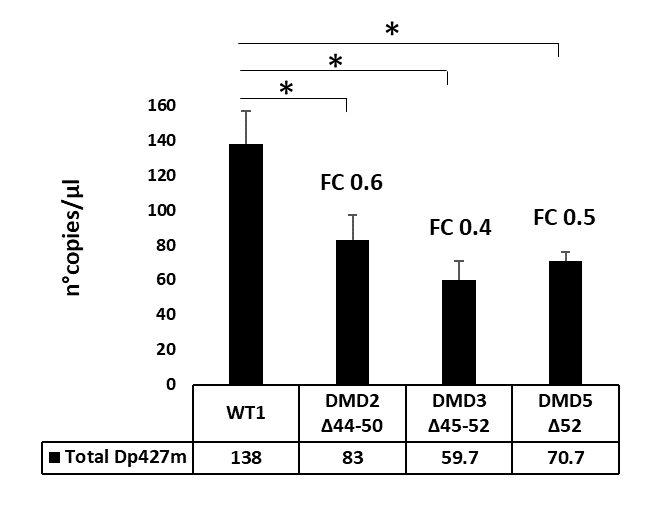
**

**Supplementary figure 2 (S2)**.

*In situ* hybridization of WT1 immortalized myoblasts using the positive probe Polr2A (RNA Polymerase II Subunit A, left panel) and the negative probe DapB (4-hydroxy-tetrahydrodipicolinate reductase, right panel). Each single Polr2A transcript is represented as a distinct red dot. DapB does not display signal. Counterstain: Gill’s Hematoxylin.


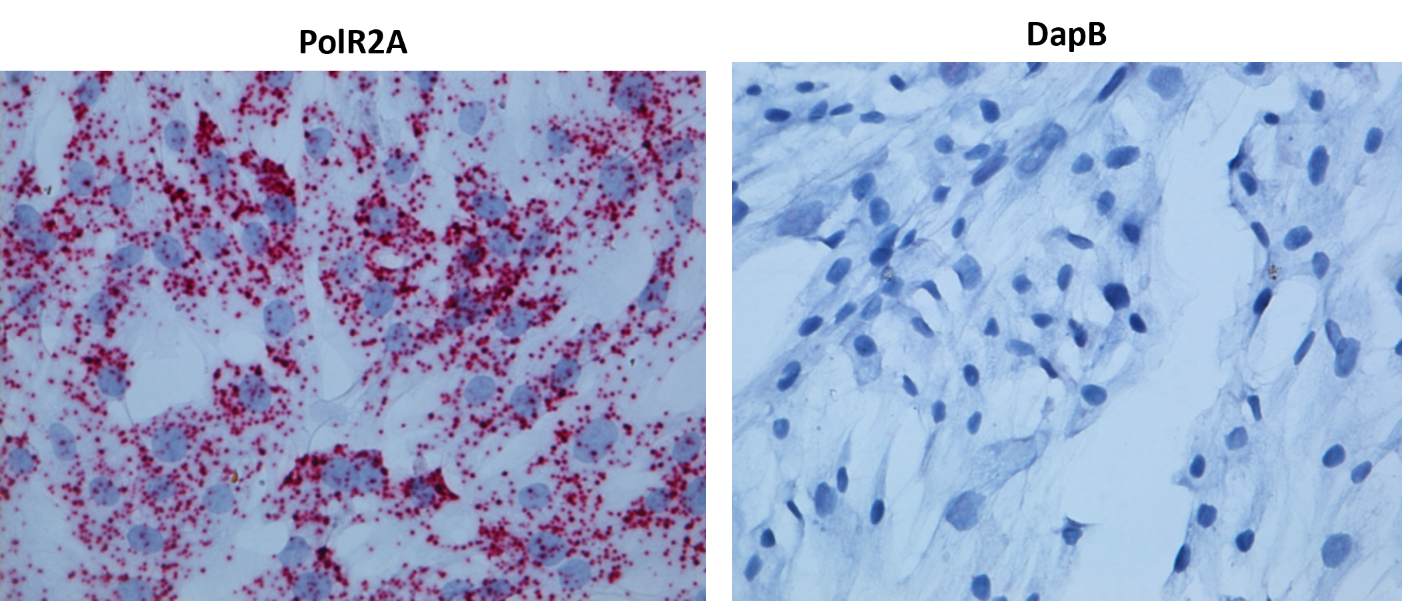


**Supplementary figure 3 (S3)**.

*DMD* transcript profile of WT and DMD patients using RNAscope.

A) The quantification of total red dots per cell shows lower dystrophin transcript levels in all DMD immortalized myoblasts compared to WT using both the 37-42 probe and the 63-75 probe.

B) DMD myotubes have lower Dp427m transcript compared to WT1 myotubes using the 37-42 probe.

We observed a high number of signals in DMD2 and DMD4 myotubes using the 63-75 probe.

C and D) The quantification of total red dots per cell (C) and per fiber (D) shows that all muscles from DMD patients have lower dystrophin transcript levels compared to WT muscle using both the 37-42 and 63-75 probes.

Bar: SEM; ** p< 0.001; * p< 0.05.


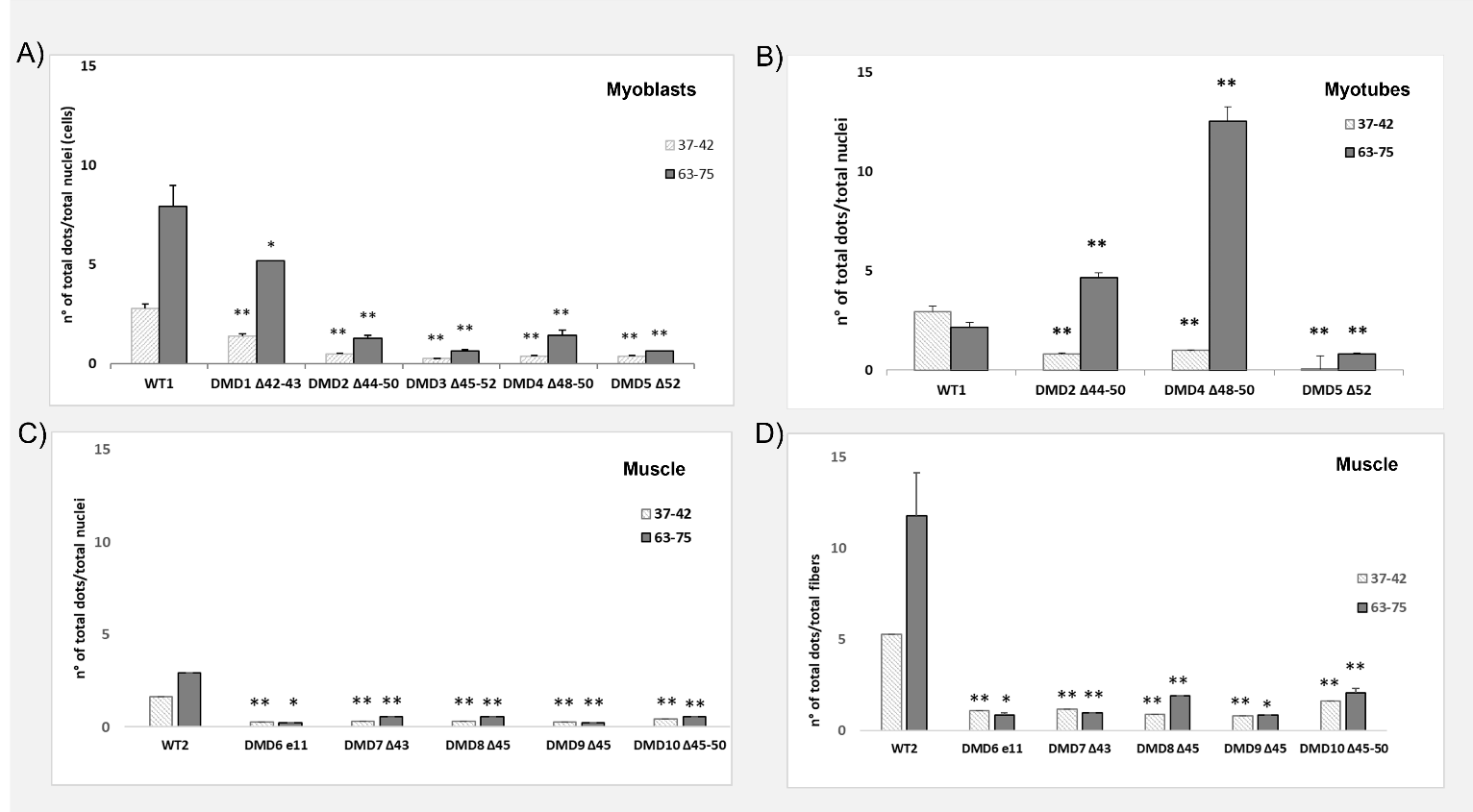


**Supplementary figure 4 (S4)**.

Expression profile of *DMD* isoforms on total mRNA from WT and DMD skeletal muscles using Real-time PCR. Dp427m is the most expressed isoform (A) and Dp71 is enriched in both WT2 and DMD compared to the other isoforms (B). Bar, standard deviation (SD).

ΔDMD represents the average of DMD6, DMD7, DMD8, DMD9, and DMD10.


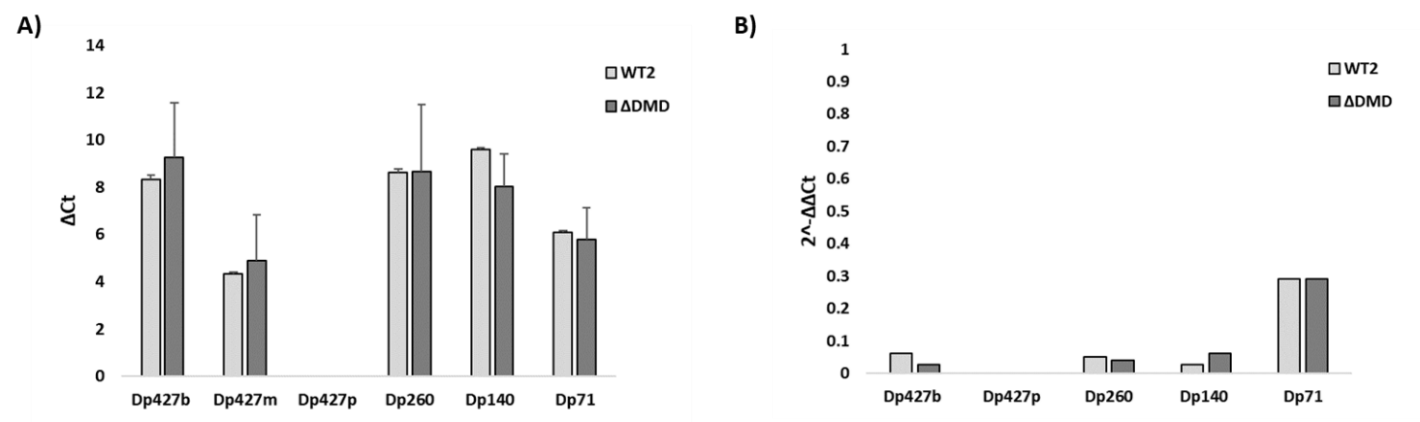


**Supplementary figure 5 (S5).**

Nuclear and cytoplasmic localization profile of *DMD* transcript in immortalized myoblasts from WT and DMD patients.

The % of dystrophin signal in nuclear or cytoplasmic compartments was calculated as a ratio of total Nuclear (N) or Cytoplasmic (C) dots and Total dots.

The different profile of *DMD* transcript localization between WT and DMD myoblasts is highlighted by the polynomial curves (dotted curve). Poly. Polynomial curve.


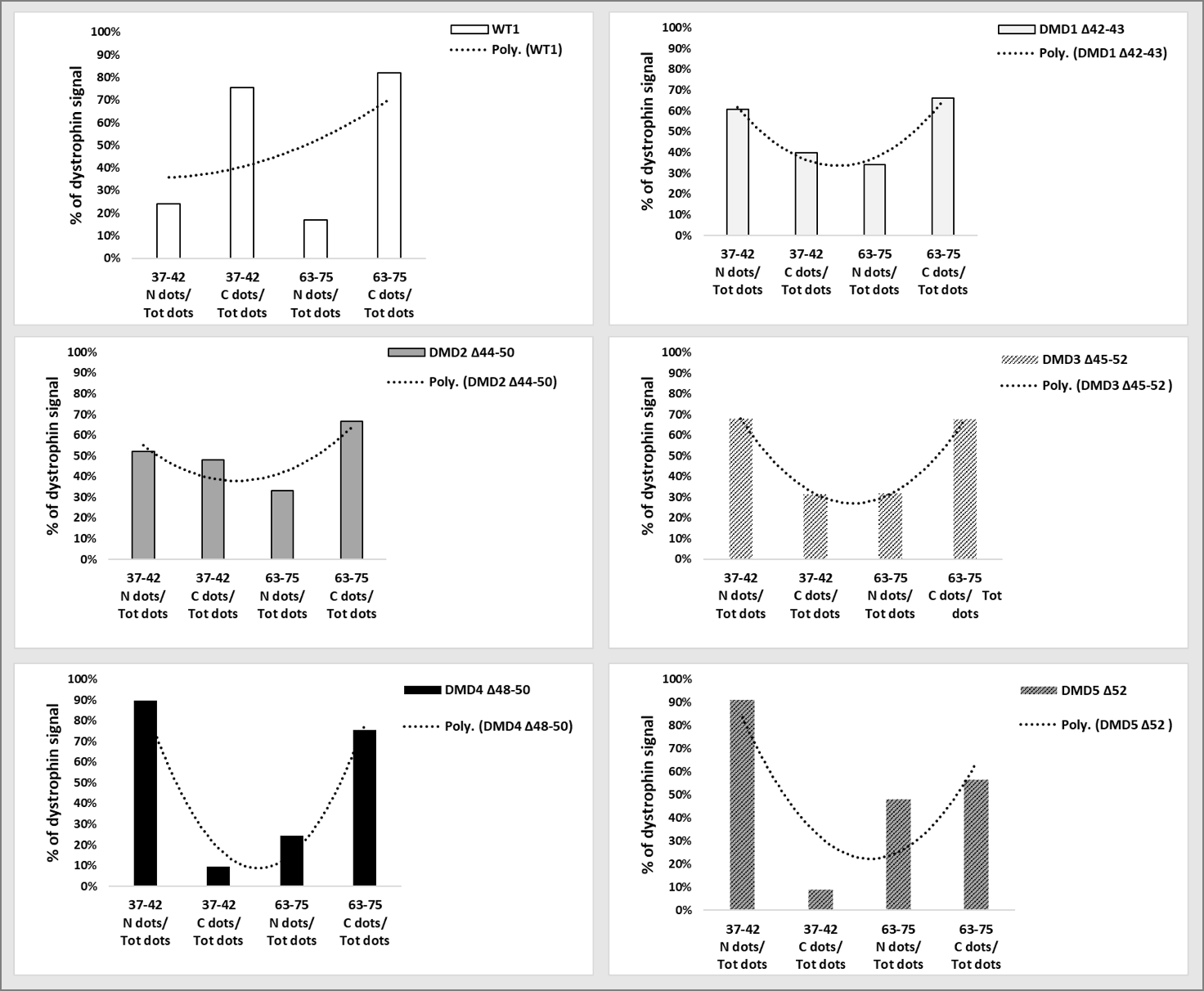


**Supplementary figure 6 (S6).**

Nuclear and cytoplasmic localization profile of *DMD* transcript in immortalized myotubes from WT and DMD patients.

The % of dystrophin signal in nuclear or cytoplasmic compartments was calculated as a ratio of total Nuclear (N) or Cytoplasmic (C) dots and Total dots.

The different profile of *DMD* transcript localization between WT and DMD myotubes is highlighted by the polynomial curves (dotted curve). Poly. Polynomial curve.


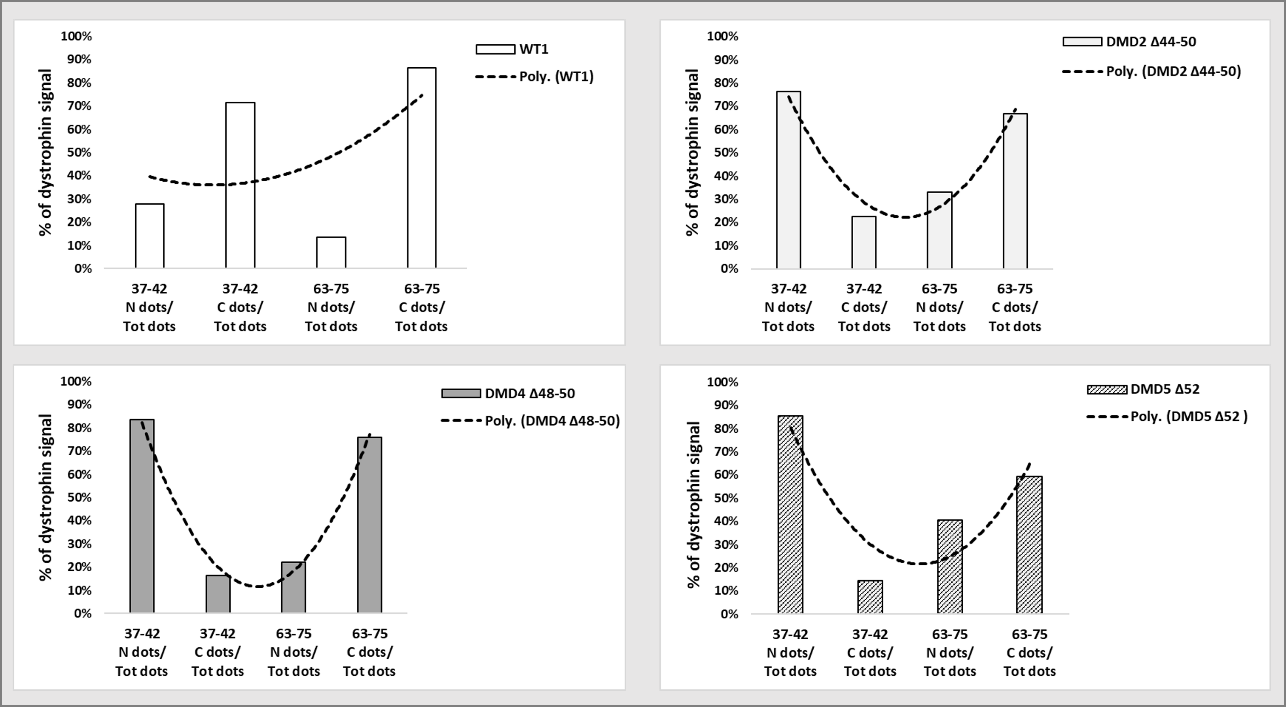


**Supplementary figure 7 (S7).**

Nuclear and cytoplasmic localization profile of *DMD* transcript in skeletal muscles (SKM) from WT and DMD patients.

The % of dystrophin signal in nuclear or cytoplasmic compartments was calculated as a ratio of total Nuclear (N) or Cytoplasmic (C) dots and Total dots.

The different profile of *DMD* transcript localization between WT and DMD skeletal muscles is highlighted by the polynomial curves (dotted curve). Poly. Polynomial curve.


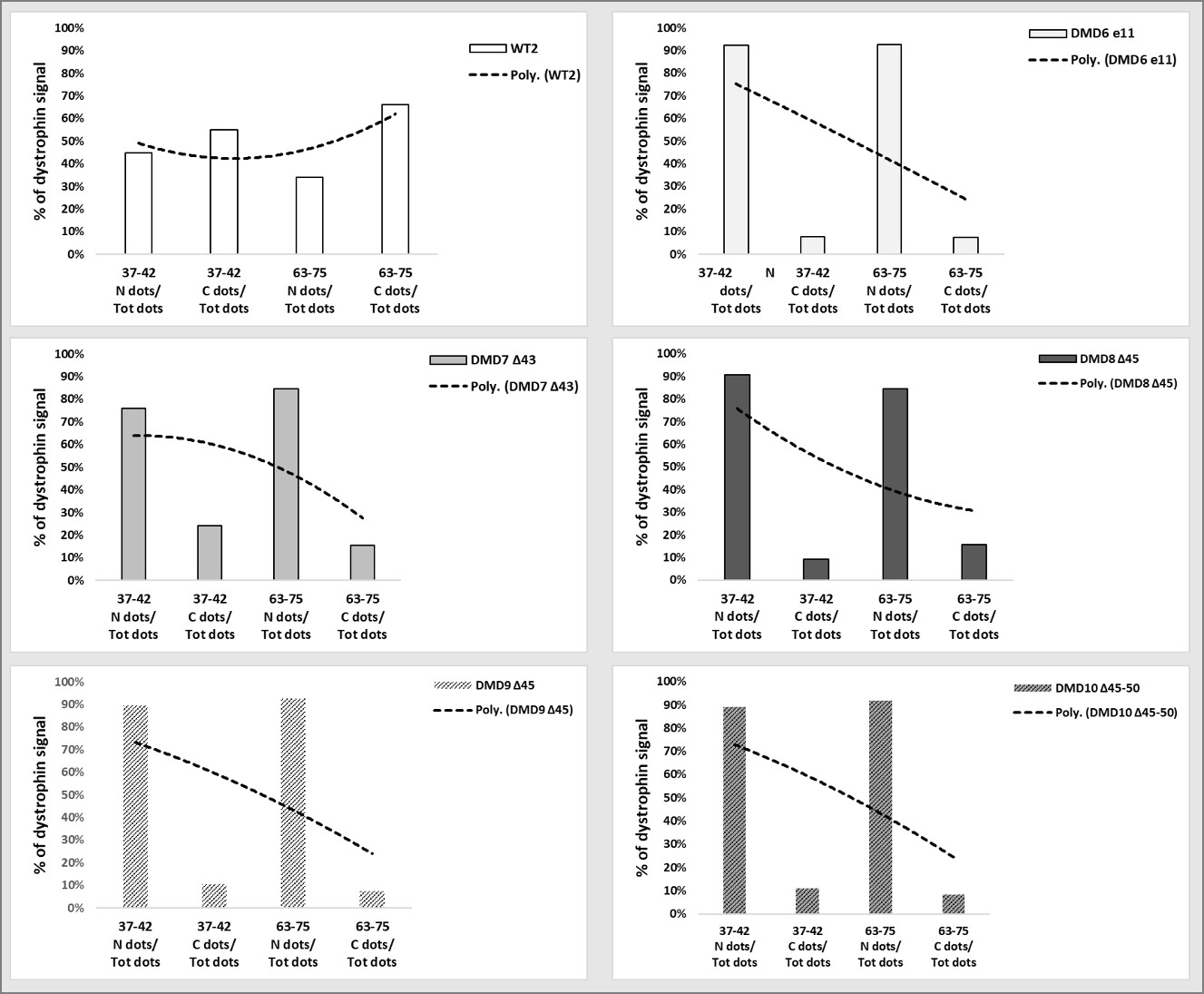


**Supplementary figure 8 (S8).**

Real-time PCR on immunoprecipitated RNA by Magna Rip.

Nucleolin protein (NCL) was immunoprecipitated (IP) with anti-nucleolin C23 MS-3 mouse monoclonal antibody in both WT1 (WT1-NCL) and DMD myoblasts (DMD1-NCL, DMD2-NCL, DMD3-NCL, and DMD5-NCL). WT1-IgG represents the mouse IgG negative control of the IP reaction.

The abundance of *DMD* isoforms in myoblasts’ RNA after either nucleolin IP or IgG IP was assessed by Real-time PCR analysis.

The left graph shows Ct values of Dp427m and Dp71 isoforms, and of RPL13A as a control of nucleolin protein immunoprecipitation, since it is a known target of nucleolin protein and therefore expected to be detected (https://doi.org/10.1093/nar/gkr488).

We observed the absence of Dp427m and Dp71 in both WT1 and DMD immunoprecipitated RNAs, while

RPL13A was immunoprecipitated in all samples.

The specific enrichment of RPL13A in IP procedure is reported on the right graph, showing fold change value of 2.15 for WT-NCL IP over the WT-IgG IP negative control.

Bar, standard deviation (SD).


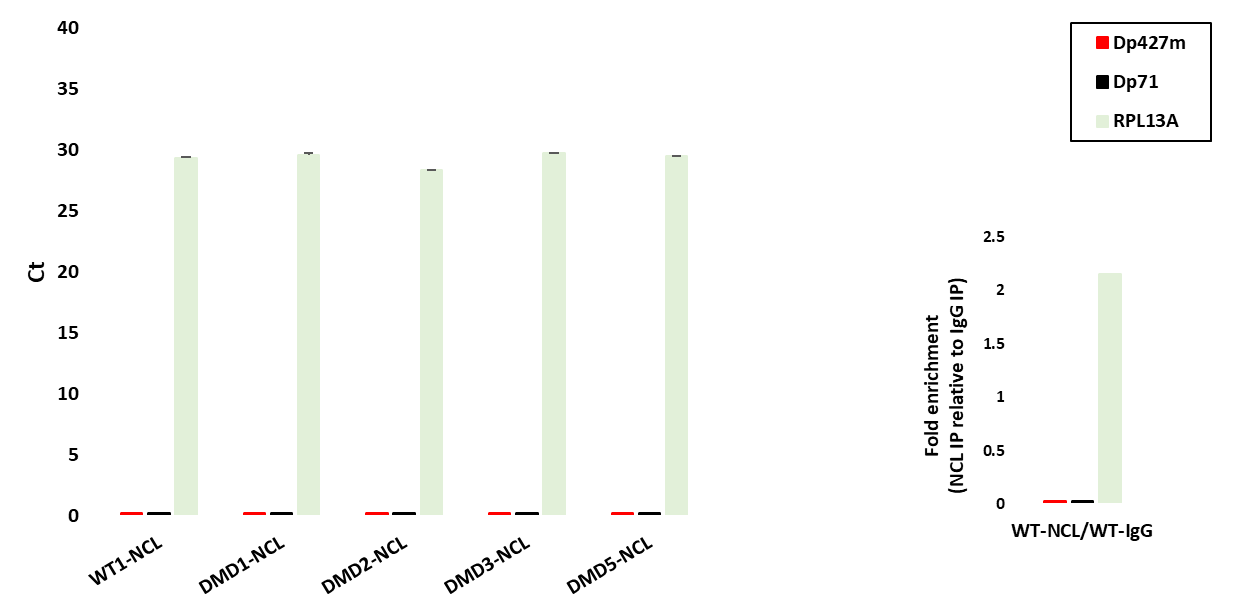


**Supplementary Table 1.**

Primers and probe sequences used in Real-time PCR and ddPCR assays.

| **NAME** | **FORWARD PRIMER SEQUENCE** | **REVERS PRIMER SEQUENCE** | **PROBE SEQUENCE** |
| --- | --- | --- | --- |
| **Dp427b** | TTGATTTGTTACAGCAGCCAACTT | CTTCCATGCCAGCTGTTTTTC | TGGCATGATGGAGTGACA |
| **Dp427m** | GAAGAACTTTTACCAGGTTTTTTTTATCG | CTTCTTCCCACCAAAGCATTTT | TGCCTTGATATACACTTTT |
| **Dp427p** | CATAGAATGTGTAAGAGAAAAGTACCAACA | GCTGGCTACACACCTTCATAGGA | AAATCAGCAAAAAGC |
| **Dp260** | AGGAAGCTGCGAAATCTGTCTT | GATGCTCTGTTCAAGCAACTTTTG | CAAAAAGGCTGTAAGGAG |
| **Dp140** | TGGCTGCTCTGAACTAAAAGCA | GCCATCCTGGAGTTCCTTAATAAG | ACCGAAAGAGGTTTTTG |
| **Dp71** | TGCAGCCATGAGGGAACAG | GGATGGTCCCAGCAAGTTGT | TCAAAGGCCACGAGACT |
